# Relationship between adult subventricular neurogenesis and Alzheimer’s disease: Pathologic roles and therapeutic implications

**DOI:** 10.3389/fnagi.2022.1002281

**Published:** 2022-09-14

**Authors:** Hyeon Soo Kim, Seong Min Shin, Sujin Kim, Yunkwon Nam, Anji Yoo, Minho Moon

**Affiliations:** ^1^Department of Biochemistry, College of Medicine, Konyang University, Daejeon, South Korea; ^2^Research Institute for Dementia Science, Konyang University, Daejeon, South Korea

**Keywords:** Alzheimer’s disease, amyloid beta, tau, adult neurogenesis, subventricular zone

## Abstract

Alzheimer’s disease (AD) is a neurodegenerative disease that is characterized by irreversible cognitive declines. Senile plaques formed by amyloid-β (Aβ) peptides and neurofibrillary tangles, consisting of hyperphosphorylated tau protein accumulation, are prominent neuropathological features of AD. Impairment of adult neurogenesis is also a well-known pathology in AD. Adult neurogenesis is the process by which neurons are generated from adult neural stem cells. It is closely related to various functions, including cognition, as it occurs throughout life for continuous repair and development of specific neural pathways. Notably, subventricular zone (SVZ) neurogenesis, which occurs in the lateral ventricles, transports neurons to several brain regions such as the olfactory bulb, cerebral cortex, striatum, and hippocampus. These migrating neurons can affect cognitive function and behavior in different neurodegenerative diseases. Despite several studies indicating the importance of adult SVZ neurogenesis in neurodegenerative disorders, the pathological alterations and therapeutic implications of impaired adult neurogenesis in the SVZ in AD have not yet been fully explained. In this review, we summarize recent progress in understanding the alterations in adult SVZ neurogenesis in AD animal models and patients. Moreover, we discuss the potential therapeutic approaches for restoring impaired adult SVZ neurogenesis. Our goal is to impart to readers the importance of adult SVZ neurogenesis in AD and to provide new insights through the discussion of possible therapeutic approaches.

## Introduction

Alzheimer’s disease (AD), the most prevalent type of dementia, is a progressive neurodegenerative disease characterized by cognitive impairment and memory loss. Two prominent neuropathological features of AD are senile plaques formed by the deposition of amyloid-β (Aβ) peptide and neurofibrillary tangles produced by the accumulation of abnormal hyperphosphorylation of tau proteins (DeTure and Dickson, 2019). Aggregation of Aβ and hyperphosphorylated tau induces various AD-associated pathologies, such as impaired adult neurogenesis (Kent et al., 2020). There has been much debate as to whether impaired adult neurogenesis contributes to the AD phenotype. Accumulating evidence suggests that the accumulation of Aβ and hyperphosphorylated tau exacerbates adult neurogenesis, and consequently, impaired adult neurogenesis induces cognitive deficits (Li Puma et al., 2020). Indeed, the elimination of adult neurogenesis worsens cognitive function (Hollands et al., 2017; Choi et al., 2018), whereas an increase in adult neurogenesis restores cognitive function in AD mouse models (Wang et al., 2010; Singh et al., 2012; Yan et al., 2014).

Neurogenesis is the process through which neural stem cells (NSCs) divide, migrate, and differentiate into specialized neural phenotypes (Cope and Gould, 2019). Adult neurogenesis progresses throughout life to consistently repair and further develop specific neural tracts (Eriksson et al., 1998; Boldrini et al., 2018), and it prominently occurs in particular regions of the neurogenic niche of the brain: the subgranular zone (SGZ) of the dentate gyrus and the subventricular zone (SVZ) bordering the lateral ventricles (Ming and Song, 2011). In a healthy brain, adult neural progenitor cells (NPCs) of the SVZ migrate through the rostral migratory stream (RMS) and eventually to the olfactory bulb (OB), where they differentiate into periglomerular and granule cells (Belluzzi et al., 2003; Arias-Carrion et al., 2007). Interestingly, several studies have shown that SVZ-derived adult NSCs and NPCs can partially migrate to the OB, cerebral cortex, striatum, and hippocampus (Seaberg and van der Kooy, 2002; Yamashita et al., 2006; Faiz et al., 2008; Motamed et al., 2019). Some evidence shows that new neurons originating from adult SVZ neurogenesis are associated with cognitive function through duplication and filling of multiple neuronal circuit regional connections within the brain (Parent et al., 1995; Bernier et al., 2002; Evans et al., 2002; Dayer et al., 2005). Surprisingly, studies examining the self-renewal ability in SVZ and dentate gyrus primary neurons have reported that self-renewal ability was found only in the SVZ (Seaberg and van der Kooy, 2002). This finding suggests that the SVZ can supply neural precursor cells to the SGZ through its continuous self-renewal capacity (Seaberg and van der Kooy, 2002), so the SVZ can be considered as the radical region of adult neurogenesis and is of comparable importance to the SGZ.

Compared with adult neurogenesis in the SGZ, few studies have focused on adult neurogenesis in the SVZ, but it is evident that the alteration of adult SVZ neurogenesis plays a crucial role in AD (Scopa et al., 2020; Culig et al., 2022). In addition, adult SVZ neurogenesis has been proposed in several studies as a promising therapeutic target for neurodegenerative diseases, including AD (Mu and Gage, 2011; Culig et al., 2022). Furthermore, SVZ-derived newborn neurons could help compensate for the reduced the neuronal population and restore impaired neural circuits in AD (Brinton and Wang, 2006). In this review, we summarize the alterations in adult SVZ neurogenesis in AD mouse models and patients with AD and suggest possible mechanisms. Furthermore, we discuss various pharmacological and non-pharmacological strategies targeting altered adult SVZ neurogenesis in AD, and describe the mechanisms of action of the three agents currently undergoing clinical trials.

## Subventricular zone neurogenesis in Alzheimer’s disease

### Alteration of adult subventricular zone neurogenesis in Alzheimer’s disease

**FIGURE 1:**
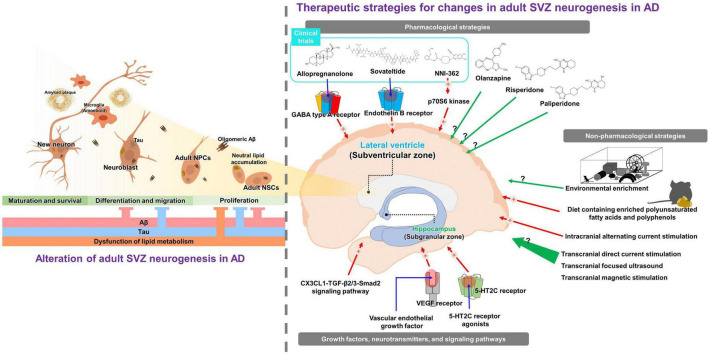
Graphical abstract for pathologic roles and therapeutic implications on adult subventricular neurogenesis in Alzheimer’s disease (AD). The left panel indicates the pathologic roles of adult subventricular neurogenesis in AD. Accumulations of Aβ, tau, and lipid inhibit proliferation, differentiation, and migration of adult neural stem cells. The right panel presents therapeutic strategies for impaired adult subventricular neurogenesis in AD described in the review. Green arrows indicate approaches known to increase adult neurogenesis in healthy animals; however, their efficacy in AD has not yet been investigated. Red arrows indicate approaches known to rescue impaired adult neurogenesis in AD.

Increasing evidence shows alterations in adult SVZ neurogenesis in patients with AD and in animal models (Figure 1 and Table 1). Notably, several studies have reported impairment of adult SVZ neurogenesis during the early stages of AD. A study examining alterations in the SVZ in an early Tg2576 mouse model reported that the proliferation of adult NSCs in the SVZ decreased from 1.5 months of age. In particular, decreased proliferation of adult NSCs is induced by intracellular Aβ oligomers (Scopa et al., 2020). Investigation of adult SVZ neurogenesis in APP/PS1 mice, which are known to exhibit Aβ deposition from 6 weeks of age and cognitive impairment at 7 months of age, showed that the proliferation and migration of NPCs decreased in the SVZ when the mice were 3 and 6 months old (Esteve et al., 2022). Studies using 2-month-old APPswe/PS1ΔE9 mice demonstrated that proliferation of NPCs and migration of neuroblasts significantly decreased in the SVZ (Demars et al., 2010). Reduced adult NSCs, NPCs, and their proliferation in SVZ were confirmed in 4-month-old 5XFAD mice (Park et al., 2016; Liu et al., 2020). In addition, the proliferation of adult NSCs was dramatically reduced in the Aβ-injected cortex compared with the contralateral cortex in Aβ-injected mice (Haughey et al., 2002). Specifically, Aβ inhibits the proliferation of adult NSCs originating from the SVZ by downregulating the expression of positive cell cycle modulators, including CDK5/p35 and cyclin B1, and upregulating the expression of the negative modulator Cdh1 (Esteve et al., 2022). Accumulated findings in an AD mouse model indicate that Aβ is a powerful factor that reduces adult SVZ neurogenesis by inhibiting the cell cycle, thereby inhibiting cell proliferation. Tau inhibits the morphological differentiation of adult NSCs into mature neurons *via* mediation of Aβ-induced microtubule hyperstabilization in Tg2576 mice (Scopa et al., 2020). The number of adult NSCs in the SVZ was reduced at 3 months of age in a 3xTg mouse model in which Aβ and tau were co-expressed (Rodriguez et al., 2009). Tauopathy mice showed a decrease in the proliferation of adult NSCs in the SVZ from 2 months of age (Komuro et al., 2015). Although tau plays a key role in the impairment of SVZ neuronal proliferation and migration under conditions such as chronic stress (Dioli et al., 2021), its effect on adult SVZ neurogenesis in AD remains unclear. Finally, dysfunction of subependymal lipid metabolism plays a critical role in the deficits of adult SVZ neurogenesis in the early stage of AD *via* hyperactivation of the protein kinase B signaling pathway (Gontier et al., 2015; Scopa et al., 2020). Decreased adult SVZ neurogenesis with neutral lipid accumulation has been reported in 2-month-old 3xTg mice (Hamilton et al., 2015), and deposition of neutral lipids was also found in the SVZ of post-mortem brains of AD patients (Hamilton et al., 2015). Similarly, proliferation in the recipient region, such as the OB, was reduced in 6 to 7-month-old ApoE4-knock-in mice (Li et al., 2009). These results suggest that the neurogenic niche in the SVZ is impaired in the early stages of AD due to Aβ and tau accumulation and disturbances in lipid metabolism.

**TABLE 1:** Alteration of adult subventricular zone neurogenesis in Alzheimer’s disease.

Decrease of adult subventricular zone neurogenesis in AD				
Animal models of AD				
Model	Method	Age (month)	Main findings	Reference
3xTg mice	IHC	2, 3, 4, 6, 9, and 12	HH3, a marker of proliferating mitosis, immunoreactivity in SVZ of 3xTg mice was decreased from 3 to 12 months of age in comparison with age-matched control.	Rodriguez et al., 2009
3xTg mice	IHC	11 and 18	Ki-67^+^/Mash-1^+^ cells in SVZ of 3xTg mice were decreased compared with age-matched control. There was no significant differences in the number of Ki-67^+^ cells between 11 and 18 months old 3xTg mice, but a decreasing trend was observed.	Hamilton et al., 2010
3xTg mice	IHC	2	Ki-67^+^ cells in SVZ of 3xTg mice were reduced than that of wild-type mice. No significant differences in DCX^+^ cells in SVZ of 3xTg mice compared with wild-type.	Hamilton et al., 2015
5XFAD mice	IHC	4	DCX^+^ cells in SVZ of 5XFAD mice were decreased compared with control.	Park et al., 2016
5XFAD mice	IHC	4	Nestin^+^, Ki-67^+^, and DCX^+^ cells in SVZ of 5XFAD mice were significantly reduced than that of wild-type.	Liu et al., 2020
APP mutant mice	IHC	11 to 12	BrdU^+^ cells in SVZ and cerebral cortex of APP mutant mice were significantly reduced compared with those in control.	Haughey et al., 2002
Aβ-injected mice	IHC	4	BrdU^+^ cells in SVZ and cerebral cortex of the Aβ-injected hemisphere were decreased compared with contralateral cortices and vehicle-injected ipsilateral SVZ.	
Tg2576 mice	IHC	1.5	Sox2^+^-positive neural stem cells were decreased in the SVZ of Tg2576 mice than that of wild-type mice. BrdU^+^ cells were decreased in the recipient regions of SVZ neurogenesis in Tg2576 mice.	Scopa et al., 2020
APP/PS1 mice	IHC	3 and 6	EdU^+^ cells were decreased in neurospheres from APP/PS1 mice. DCX^+^ and NeuN^+^ cells were decreased in RMS of APP/PS1 mice.	Esteve et al., 2022
APP/PS1 mice	IHC	2	DCX^+^ and BrdU^+^ cells of SVZ were significantly decreased in APPswe/PS1ΔE9 mice compared with wild-type mice.	Demars et al., 2010
hTau mice	IHC	2, 6, and 12	Ki-67^+^ cells in SVZ of hTau mice were reduced from 2 months of age.	Komuro et al., 2015
ApoE4-KI mice	IHC	6 to 7	BrdU^+^ cells were decreased in the recipient region of SVZ neurogenesis in ApoE4-KI mice.	Li et al., 2009

**AD patients/AD patient-derived cell**				
**Subjects**	**Method**	**Ages (year)**	**Main findings**	**Reference**
AD patients	IHC	82.50 ± 4.97	NPCs of SVZ were decreased in AD patients compared with control.	Ziabreva et al., 2006
MCI AD patients	Multimodal MRI	MCI: 66.8 ± 6.0 AD: 68.7 ± 7.8	Region-specific increased mean diffusivity values in SVZ.	Cherubini et al., 2010
AD patients	IHC	81.2 ± 7.0	Musashi-1^+^ cells decrease with progressing Braak stages in AD patients.	Perry et al., 2012
**Increase of adult subventricular zone neurogenesis in AD**				
**Animal models of AD**				
PDGF-APP mice	IHC	3 and 12 months	DCX^+^ cells in SVZ of PDGF-APP mice was not significantly changed at 3 months of age but increased at 12 months.	Jin et al., 2004
**AD patients/AD patient-derived cell**				
AD patients	WST-1 assay	86.9 ± 1.1	NPCs of SVZ are increased in AD patients compared with the healthy group.	Mastroeni et al., 2016

AD, Alzheimer’s disease; Aβ, Amyloid beta; IHC, Immunohistochemistry; MCI, Mild cognitive impairment; NPCs, Neural progenitor cells; NSCs, Neural stem cells; WB, Western blot; BrdU, Bromodeoxyuridine; DCX, Doublecortin; PSA-NCAM, Polysialylated-neural cell adhesion molecule; EdU, 5-Ethynyl-2’-deoxyuridine; RMS, Rostral migratory stream; OB, Olfactory bulb; HH3, histone H 3.

As AD progresses, the increased interaction between Aβ and tau induces synergistic neurotoxicity (Zhang et al., 2021). Several other factors, along with Aβ and tau, strongly inhibit adult neurogenesis during the progression of AD. In this respect, dysregulations of inflammatory factors such as IL-6 and factors contributing to protein homeostasis may contribute to the impairment of adult SVZ neurogenesis in neurodegenerative disease (Wang et al., 2016). One study showed that adult NPC proliferation was significantly reduced by Aβ-induced dysregulation of calcium homeostasis in the SVZ of 11 to 12-month-old APP mutant mice (Haughey et al., 2002). Moreover, 7-month-old 5XFAD mice had higher Aβ deposition and microgliosis in the cerebral cortex than those in 2-month-old 5XFAD mice. SVZ-derived NPC from these 7-month-old 5XFAD mice exhibited impairments in general functions, including expansion of the neurosphere, immunomodulation, neurotrophic regulation, recovery from neurotoxic stimuli, and migration to the defective region (Fainstein et al., 2018). Adult SVZ neurogenesis, such as generating proliferating and progenitor cells, considerably decreased with AD progression in 11-and 18-month-old 3xTg mice compared with that in 2-month-old 3xTg mice (Hamilton et al., 2010, 2015). Consistent with the AD animal model, alterations in adult neurogenesis have also been reported in the brains of AD patients (Moreno-Jimenez et al., 2019). A post-mortem study of patients with AD showed that adult NPCs were reduced compared to those in healthy controls (Ziabreva et al., 2006). Levels of adult NPCs decrease with progressing Braak stages in AD patients (Perry et al., 2012). An *in vitro* study on human cortical NPCs showed that Aβ can inhibit NPC proliferation and differentiation and induce apoptosis even by disrupting calcium homeostasis (Haughey et al., 2002). In addition, a multimodal magnetic resonance imaging study performed on MCI and AD patients suggests that adult SVZ neurogenesis may be continuously impaired in MCI (Cherubini et al., 2010). Many studies have demonstrated that adult SVZ neurogenesis deteriorates due to various complex factors, including calcium homeostasis dysfunction and microgliosis, during AD progression. Therefore, approaches that target the impairment of adult SVZ neurogenesis are important in both asymptomatic and symptomatic stages of AD.

### Therapeutic strategies for changes in adult subventricular zone neurogenesis in Alzheimer’s disease

Adult neurogenesis is important in compensating for neuronal loss and restoring cognitive function. Various pharmacological and non-pharmacological strategies have been investigated to enhance or restore adult neurogenesis (Figure 1). Several antipsychotic drugs have been reported to increase adult neurogenesis in the SVZ. One study has shown that antidepressants, such as olanzapine, increase adult SVZ neurogenesis (Green et al., 2006). In addition, administration of atypical neuroleptics, such as risperidone and paliperidone, increases adult SVZ neurogenesis in rats (Wakade et al., 2002; Nasrallah et al., 2010). It has been shown that, like pharmacological strategies, non-pharmacological therapeutic strategies can restore SVZ neurogenesis in AD. Environmental reinforcement is known to upregulate both adult SVZ and SGZ neurogenesis (van Praag et al., 1999; Mastrorilli et al., 2017). Although environmental enrichment enhances adult hippocampal neurogenesis in APP23 and APP_*Sw, Ind*_ mice (Mirochnic et al., 2009; Valero et al., 2011), few studies have reported the effect of environmental enrichment on adult SVZ neurogenesis. In apoE4-transgenic mice, environmental enrichment only increased adult neurogenesis in the SGZ (Levi and Michaelson, 2007). Further studies are needed to determine the effects of environmental enrichment on adult SVZ neurogenesis in other AD transgenic models. In addition, the LMN diet, a diet containing enriched polyunsaturated fatty acids and polyphenols, increased adult SVZ neurogenesis and alleviated cognitive impairment in Tg2576 mice (Fernandez-Fernandez et al., 2012). Intracranial alternating current stimulation has been reported to increase neurogenesis in the adult SVZ and SGZ in 5XFAD mice (Liu et al., 2020). Although there are an increasing number of studies in which non-invasive stimulation, such as transcranial direct current stimulation, transcranial focused ultrasound, and transcranial magnetic stimulation, increases adult neurogenesis in normal mice (Ueyama et al., 2011; Scarcelli et al., 2014; Pikhovych et al., 2016), there have been no investigations into whether these stimulations rescue impaired adult neurogenesis in AD.

Adult SVZ neurogenesis is modulated by various growth factors, neurotransmitters, and signaling pathways. Serotonin neurotransmission is important in the early stages of SVZ cell proliferation because serotonin (5-HT) terminals play an important role in forming the dense plexus that regulates ventricle and type B1 cells (Banasr et al., 2004; Tong et al., 2014). In particular, the level of the serotonin receptors decreased in the brains of patients with AD (Lorke et al., 2006). In practice, stimulation of 5-HT2C receptors *via* the administration of agonists increases adult SVZ neurogenesis in rats (Banasr et al., 2004). A strategy to modulate the level of vascular endothelial growth factor (VEGF) has also been proposed as a promising approach for restoring impaired neurogenesis (Sawada et al., 2014). The role of VEGF in adult neurogenesis in the neurogenic niche, including the SVZ, is well-established (Jin et al., 2002; Sawada et al., 2014). Implantation of VEGF through nanospheres effectively increased adult neurogenesis in APP/PS1 mice (Antequera et al., 2012; Herran et al., 2015). Finally, modulating activation of the signaling pathways involved in adult neurogenesis can enhance neurogenesis in the SVZ. For instance, stimulation of the CX3CL1-TGF-β2/3-Smad2 signaling pathway increases adult SVZ neurogenesis and ameliorates tau pathology-induced cognitive decline (Fan et al., 2019, 2020).

Three pharmaceutical candidates targeting adult neurogenesis are currently undergoing clinical trials for the treatment of AD (Cummings et al., 2022). Allopregnanolone is a neurosteroid that promotes adult SVZ neurogenesis by modulating GABA type A receptors (Wang et al., 2005; Brinton, 2013; Karout et al., 2016). Interestingly, allopregnanolone significantly decreased in brains with AD (Weill-Engerer et al., 2002). Studies in the 3xTg mouse model demonstrated that allopregnanolone increased adult neurogenesis in the SVZ and SGZ, and significantly recovered cognitive function in 3xTg mice (Wang et al., 2010; Singh et al., 2012). Moreover, a relevant study revealed that allopregnanolone stimulates NPC proliferation (Wang et al., 2005; Wang and Brinton, 2008). These results suggest that allopregnanolone promotes adult SVZ neurogenesis in AD and may be a therapeutic agent for AD. Allopregnanolone is currently in phase 1 and 2 clinical trials (NCT03748303 and NCT 04838301). Second, sovateltide (IRL-1620 or PMZ-1620) is an endothelin B receptor (ETBR) agonist that promotes adult SVZ neurogenesis. Previous studies have shown that the ETBR system is involved in neuronal cell survival and the restoration of adult neurogenesis in neurodegenerative disease (Leonard et al., 2011, 2012; Ranjan et al., 2020). Moreover, some studies have revealed that elevated levels of ETBR in AD are correlated with Aβ levels (Palmer et al., 2009; Koyama, 2013). In addition, a study in Aβ-injected rats suggested that sovateltide, an ETBR agonist, restored adult neurogenesis by promoting nerve growth factor and VEGF (Briyal et al., 2015). Furthermore, treatment of Aβ-injected rats with sovateltide significantly restored cognitive function (Briyal et al., 2014). Based on these neurogenesis-related effects, sovateltide for AD is undergoing a phase 2 clinical trial (NCT 04052737). NNI-362 is a small molecule that enhances adult SGZ neurogenesis by stimulating allosteric p70S6 kinase (Sumien et al., 2021). In addition, the administration of NNI-362 significantly restored cognitive function in Ts65Dn mice (Sumien et al., 2021). NNI-362 is a phase 1 clinical trial for AD (NCT 04074837). Unfortunately, NNI-362 is restricted to adult SGZ neurogenesis, and its effect on adult SVZ neurogenesis has not yet been confirmed. Although adult neurogenesis compensates for neuronal loss and restores cognitive function, studies, and clinical trials targeting AD are limited.

## Discussion

In this review, we summarize the changes in adult SVZ neurogenesis in AD mouse models and patients with AD. Furthermore, we describe the possibility of therapeutic approaches to target altered adult SVZ neurogenesis in AD. Since SVZ-derived neuronal cells can migrate to the neocortex, striatum, hippocampus, and OB to compensate for neuronal loss, stimulating adult SVZ neurogenesis may provide a potential benefit in the restoration of impaired cognitive function in AD (Yamashita et al., 2006; Rodriguez et al., 2009; Rodriguez and Verkhratsky, 2011). In addition, adult SVZ neurogenesis is impaired from an early stage of AD and may contribute to the onset and progression of AD *via* various mechanisms (Rodriguez et al., 2009; Scopa et al., 2020). In particular, the striatum, which receives neuronal cells originating from the SVZ, is important for cognitive functions, including learning and memory, and neuronal loss in the ventral tegmental area is associated with memory dysfunction in AD (Ernst et al., 2014; Nobili et al., 2017). Therefore, alterations in adult SVZ neurogenesis have important implications as promising therapeutic targets in AD.

Despite many studies reporting the impairment of adult SVZ neurogenesis in AD, few studies have shown increased adult SVZ neurogenesis in AD (Table 1; Jin et al., 2004; Mastroeni et al., 2016). It can be speculated that Aβ could transiently induce hormetic effects in adult neurogenesis at subthreshold concentrations that induce neurotoxicity in early AD (Kent et al., 2020). For instance, Aβ-induced neurogenic effects in hippocampal NSCs are caused exclusively by Aβ_42_ but not by Aβ_25_–_35_ or Aβ_40_, and these effects are mediated by the extracellular signal-regulated kinase (ERK) pathway (Lopez-Toledano and Shelanski, 2004). Aβ_42_ is involved in adult neurogenesis by increasing DNA polymerase-β (Calafiore et al., 2012). Furthermore, adult SVZ neurogenesis could be temporarily enhanced as a compensatory response to massive nerve loss and brain injury during AD progression (Jin et al., 2004; Mu and Gage, 2011). Although adult SVZ neurogenesis is an attractive therapeutic target for AD, further studies are required. First, the migration pathways of SVZ-derived immature neurons in the human brain are not fully understood. Although the migration of neuroblasts has been observed in animals, the presence of RMS and OB neurogenesis in humans is controversial (Arellano and Rakic, 2011; Sanai et al., 2011; Bergmann et al., 2015). In addition, more studies are required to determine the pathological implications of tau in adult SVZ neurogenesis in AD. Although some studies have reported the inhibitory effects of tau pathology on adult SVZ neurogenesis (Rodriguez et al., 2009; Komuro et al., 2015; Scopa et al., 2020), the mechanisms of tau pathology in adult SVZ neurogenesis in the AD brain are still limited. Interspecies differences in adult SVZ neurogenesis between humans and mice should also be considered when interpreting the results obtained from AD mouse models (Lazarov and Marr, 2013). Thus, more mechanistic and translational studies on adult SVZ neurogenesis in AD should be performed.

## Author contributions

HK, SS, SK, and YN drafted the initial version of the manuscript. AY conceived the tables. YN made the figure. MM conceived the outline of the manuscript, provided critical input, and revised the initial version of manuscript. All authors approved the final version of the manuscript.
